# Spatial microbiome-metabolic crosstalk drives CD8^+^ T-cell exhaustion through the butyrate-HDAC axis in colorectal cancer

**DOI:** 10.3389/fmicb.2025.1704491

**Published:** 2025-12-08

**Authors:** Xiaoyang Chen, Yinxu Zhang, Guangyu Zhang, Dai Wang, Linhua Dou, Yuxi Wang, Zining Huang, Xiaomei Liu

**Affiliations:** 1 Department of Oncology, The First Affiliated Hospital of Jinzhou Medical University, Jinzhou, Liaoning, China; 2 Department of Surgery, The First Affiliated Hospital of Jinzhou Medical University, Jinzhou, Liaoning, China; 3 Department of Anesthesiology, Medical College of Jinzhou Medical University, Jinzhou, Liaoning, China; 4 Huashan School of Medicine, Shanghai Medical College, Fudan University, Shanghai, China

**Keywords:** spatial microbiome, butyrate metabolism, CD8^+^ T-cell exhaustion, HDAC inhibition, microbiome-metabolic-immune crosstalk, colorectal cancer

## Abstract

**Background:**

The spatial organization of intratumoral microbiota and its metabolic impact on immunotherapy response in colorectal cancer (CRC) is unclear, limiting targeted interventions.

**Methods:**

We integrated single-cell RNA-seq, spatial transcriptomics, and microbial multi-omics from a discovery cohort of 23 treatment-naïve CRC patients. Findings were validated in an independent validation cohort from The Cancer Genome Atlas (TCGA-CRC, *n* = 159).

**Results:**

Spatial depletion of *Streptococcus* and *Acetivibrio* in tumor niches disrupts butyrate-histone deacetylase (HDAC) signaling, leading to programmed cell death 1 (PDCD1) hyperacetylation and CD8^+^ T-cell exhaustion. The Colorectal Cancer Microbiome Score (CMS) may serve as a predictive biomarker for immunotherapy response and HDAC inhibitor-based combination therapy. We developed the CMS, a spatial biomarker that stratifies patients by microbial-metabolic dysfunction, predicting immunotherapy resistance (e.g., higher tumor immune dysfunction and exclusion (TIDE) scores; *p* < 0.01) and guiding combinatorial HDAC inhibition for CMS-defined subgroups. *In silico* fecal microbiota transplantation (FMT) validated CMS as an actionable target for microbiota modulation. Butyrate supplementation *in vitro* restored HDAC activity and reduced PD-1 expression on CD8^+^ T cells, validating the proposed mechanism.

**Conclusion:**

Our study unveils a spatially defined, microbiome-driven metabolic niche that epigenetically programs CD8^+^ T-cell exhaustion via the butyrate-HDAC axis, revealing a targetable mechanism to overcome immunotherapy resistance in CRC.

## 1 Introduction

Colorectal cancer (CRC) is a leading cause of cancer-related mortality worldwide (Morgan et al., 2023; Matsuda et al., 2025; Baidoun et al., 2021). The gut microbiome has emerged as a critical modulator of tumor progression, immune evasion, and therapeutic response (Hou et al., 2022; Bredin and Naidoo, 2022; Al-Qadami et al., 2022). However, the spatial distribution and localized functional impact of intratumoral microbial communities on metabolic reprogramming and immune evasion are still largely unexplored (Galeano Niño et al., 2022). Notably, Nejman et al. (2020) demonstrated that human tumors harbor tumor-type-specific intracellular bacteria, primarily within cancer and immune cells, providing a cellular basis for microbiome-mediated regulation of the tumor microenvironment. Building on this foundation, our study employs spatially resolved metagenomic and transcriptomic analyses to investigate the functional crosstalk between CRC-associated microbes and host cells, offering new insights into microbiome-driven mechanisms of metabolic reprogramming and immune evasion.

The spatial distribution and functional impact of intratumoral microbial communities within metabolic and immune niches remain poorly understood, hindering targeted therapeutic strategies. By mapping microbial-metabolic niches, CMS identifies patients likely to benefit from HDAC inhibition combined with microbiome remodeling [e.g., fecal microbiota transplantation (FMT)], addressing a critical unmet need in CRC precision oncology. From these analyses, we developed the CMS, a novel spatial biomarker designed to stratify patients based on microbial-metabolic dysfunction. To test this, we first mapped microbial-metabolic niches in a discovery cohort (*n* = 23) and then validated our findings in the TCGA-CRC cohort (*n* = 159). Notably, the discovery cohort was designed to identify candidate microbial-metabolic signatures with high biological specificity, while the large-scale TCGA-CRC validation (*n* = 159) ensured generalizability. To address potential sample size limitations in the discovery phase, we employed bootstrapping resampling and leave-one-out cross-validation, which demonstrated robust classification performance (AUC = 0.92, 95% CI: 0.88–0.96) even with the smaller cohort. Additionally, sensitivity analyses confirmed that the CMS signature remained stable across subsamples of the discovery cohort, further supporting the reliability of our findings despite the initial sample size. Our spatially resolved approach moves beyond bulk-level associations to identify niche-specific mechanisms of immune evasion, offering actionable targets for microbiome-based therapeutic interventions.

## 2 Data and analysis methods

### 2.1 Data sources

Microbial abundance profiling was conducted through re-analysis of raw RNA-seq data from TCGA-CRC samples. Quality-controlled reads were aligned against a combined reference database comprising the human genome (GRCh38) and microbial genomes using Kraken2 (v2.1.2) with default parameters. To achieve accurate genus-level abundance estimates, Bracken (v2.6.1) was employed with a minimum read threshold of 10 reads per taxon. Taxa with a prevalence below 10% across all samples were excluded to ensure robust downstream analyses. Microbial abundance data were obtained from TCGA, while RNA-seq data and survival information for the TCGA-CRC training set were sourced from UCSC Xena (Zhou et al., 2023). Single-cell RNA-seq data were acquired from GEO (GSE132465), and spatial transcriptomics data from GSE225857. Metabolic pathway gene sets were retrieved from the KEGG database (release 2025-07). For validation, transcriptomic and microbial data from an independent TCGA-CRC cohort (*n* = 159) were downloaded from UCSC Xena. Sample inclusion criteria were (1) availability of paired transcriptomic and microbial abundance data; (2) complete clinical survival information; and (3) exclusion of samples with >25% missing values in key variables.

### 2.2 Data analysis

#### 2.2.1 Single-cell transcriptome data processing

Single-cell RNA-seq data were processed using Seurat (v5.2.0). Quality control was performed by filtering out cells with fewer than 200 genes or mitochondrial content exceeding 20%. Data normalization was conducted using the “LogNormalize” method with a scale factor of 10,000, followed by scaling and identification of the top 2,000 highly variable genes for downstream analysis. The top 30 principal components (PCs) were selected based on elbow plot analysis and used for non-linear dimensionality reduction (UMAP) and cell clustering (FindClusters function, resolution = 0.3). To mitigate batch effects, Harmony (v0.1.1) was applied for integration across samples. The sample size (*n* = 23 patients) was determined by power analysis (*β* = 0.8, *α* = 0.05) based on microbial heterogeneity estimates from Galeano Niño et al. (2022). Cell type annotation was performed using well-established marker genes listed in Supplementary Table S1.

#### 2.2.2 Co-localization analysis of spatial transcriptomics and single-cell data

To enable spatially resolved mapping of microbial abundance, we developed an innovative adaptation of the Robust cell type decomposition (RCTD) algorithm (v2.2.0). Specifically, genus-level microbial abundance profiles derived from TCGA were treated as reference “cell type” profiles, leveraging RCTD’s probabilistic framework to deconvolve microbial signals onto spatial transcriptomics spots. This approach infers the spatial localization of specific microbial genera (e.g., *Streptococcus*) by modeling their distributions across tissue regions, accounting for platform effects and technical variations between reference and spatial data. The deconvolution results were integrated with unsupervised clustering (UMAP and FindClusters, resolution = 0.3) to correlate microbial spatial patterns with host gene expression gradients and immune cell distributions, providing insights into microbiome-tumor microenvironment crosstalk.

#### 2.2.3 Spatial region enrichment analysis

Marker genes for major cell compartments (immune, cancer epithelial, and stromal) were selected, including CD3D, CD4, and CD8A (immune cells); EPCAM and KRT19 (cancer epithelial cells); and ACTA2 and FAP (stromal cells). These marker genes are well-characterized in the field of colorectal cancer and single-cell biology, retrieved using the CellMarker2.0 database (Hu et al., 2023), ensuring the specificity and reliability of our spatial region enrichment analysis. AddModuleScore function calculated the feature scores. FindAllMarkers function identified highly variable genes between the tumor and adjacent regions. The compareCluster function performed enrichment analysis to screen metabolic pathways.

#### 2.2.4 Prognostic microorganism analysis

A total of 159 patients with prognostic data from the TCGA dataset were selected. Microbes with >25% missing abundance data were excluded. This threshold was chosen to balance data integrity and microbial representation: a 25% missingness cutoff is a widely accepted criterion in microbiome research to filter out taxa with insufficient data for reliable statistical analysis (Cao et al., 2020; Smirnova et al., 2019), while retaining most biologically relevant microbes. Excluding taxa with high missingness prevents bias in downstream analyses (e.g., correlation and survival modeling) and ensures the robustness of our findings. The surv_cutpoint function determined optimal cut-off values, and the logrank test identified key microbes. The GSVA package calculated CMS scores. This enabled us to correlate microbial genera (e.g., *Streptococcus*) with spatial patterns of gene expression and immune cell infiltration.

#### 2.2.5 CMS score group analysis

CMS integrates six prognostic genera (*Streptococcus*, *Acetivibrio*, *Filifactor*, *Eggerthella*, *Dorea*, and *Mediterranea*) with butyrate metabolism genes (KEGG pathway hsa00650) via GSVA, validated in TCGA-CRC (*n* = 159). The CMS score was computed by integrating the abundances of six prognostic genera and the activity of butyrate metabolism genes (KEGG pathway hsa00650) using gene set variation analysis (GSVA).

#### 2.2.6 Immune characteristic analysis

The top 100 characteristic genes were screened from single-cell data. Immune infiltration (ssGSEA), tumor microenvironment (TME) scores (ESTIMATE/IPS), and immunotherapy response (TIDE) were analyzed to evaluate CMS-associated immune dysfunction. The Wilcox method calculated differences in immune checkpoints. Specifically, we used the Wilcoxon rank-sum test (a non-parametric method) to compare the expression levels of immune checkpoint genes (e.g., PDCD1 and CTLA4) between high-CMS and low-CMS groups. This test was chosen due to the non-normal distribution of immune checkpoint expression data, and a two-tailed *p*-value <0.05 was considered statistically significant. For multiple comparisons, the Benjamini–Hochberg method was applied to control the false discovery rate (FDR), with an adjusted *p*-value (FDR) ≤0.05 deemed significant.

#### 2.2.7 IC_50_ analysis of immunochemotherapy drugs

To investigate potential therapeutic implications of the tumor microbiome, drug sensitivity (half-maximal inhibitory concentration, IC_50_) was predicted for immunochemotherapy and targeted agents (including BX-795, GDC-0941, BIBW2992, and AKT inhibitor VIII) using the pRophetic R package (Geeleher et al., 2014a; Geeleher et al., 2014b). This computational approach leverages ridge regression to model the relationship between baseline gene expression in the Cancer Genome Project (CGP) cell line panel and drug response data, subsequently applying this model to infer sensitivity in our patient-derived transcriptomic profiles (Geeleher et al., 2014b; Liu et al., 2020). The analysis was specifically focused on compounds with known immunomodulatory or microbiota-interaction potential to prioritize biologically relevant candidates (Lu et al., 2024).

#### 2.2.8 Cell communication and pseudotime analysis

The CellChat package analyzed cell–cell communication (Jin et al., 2021). The Monocle package was used for pseudotime analysis of single-cell data (Gulati et al., 2020).

#### 2.2.9 Transcription factor analysis

To elucidate host transcriptional responses potentially linked to microbial presence, gene regulatory networks were reconstructed using pySCENIC (v0.12.1) (Aibar et al., 2017; Kumar et al., 2021). This analysis identified key transcription factors (TFs), and the regulon sets of target genes were directly regulated via motif binding, whose activity may be influenced by tumor–microbiome interactions. The pySCENIC workflow was applied to our single-cell or spatial transcriptomics data as follows: (i) inference of co-expression modules, (ii) refinement of direct targets through cis-regulatory motif analysis (cisTarget databases), and (iii) calculation of regulon activity scores (AUCell) for each cell or spot (Aibar et al., 2017). This approach provided a high-resolution map of TF activity states across the tissue microenvironment, enabling correlation with spatially mapped microbial distributions.

#### 2.2.10 Statistical analysis

Statistical analyses were performed using the R language. For comparisons of continuous variables (e.g., gene expression, metabolic scores, and IC_50_ values) between two independent groups (e.g., high-CMS vs. low-CMS), the Wilcoxon rank-sum test (Mann–Whitney *U* test) was applied. A two-tailed *p*-value of less than 0.05 was considered statistically significant. For all multiple hypothesis testing scenarios (e.g., differential expression analysis across thousands of genes), the Benjamini–Hochberg (BH) procedure was used to control the false discovery rate (FDR), with an adjusted *p*-value (FDR) of ≤0.05 deemed significant.

#### 2.2.11 Ethical statement

Our research fully complies with TCGA and GEO ethical guidelines. Written approval was obtained from all participants, and the study adheres to the Declaration of Helsinki. Additionally, this study was approved by the Institutional Review Board of The First Affiliated Hospital of Jinzhou Medical University (Approval Number: KYLL202578). The research strictly adheres to the Declaration of Helsinki, ensuring the protection of participant privacy and data integrity throughout the research process.

## 3 Results

### 3.1 Analysis of cell characteristics in the single-cell transcriptomic profile of colorectal cancer

**Figure 1:**
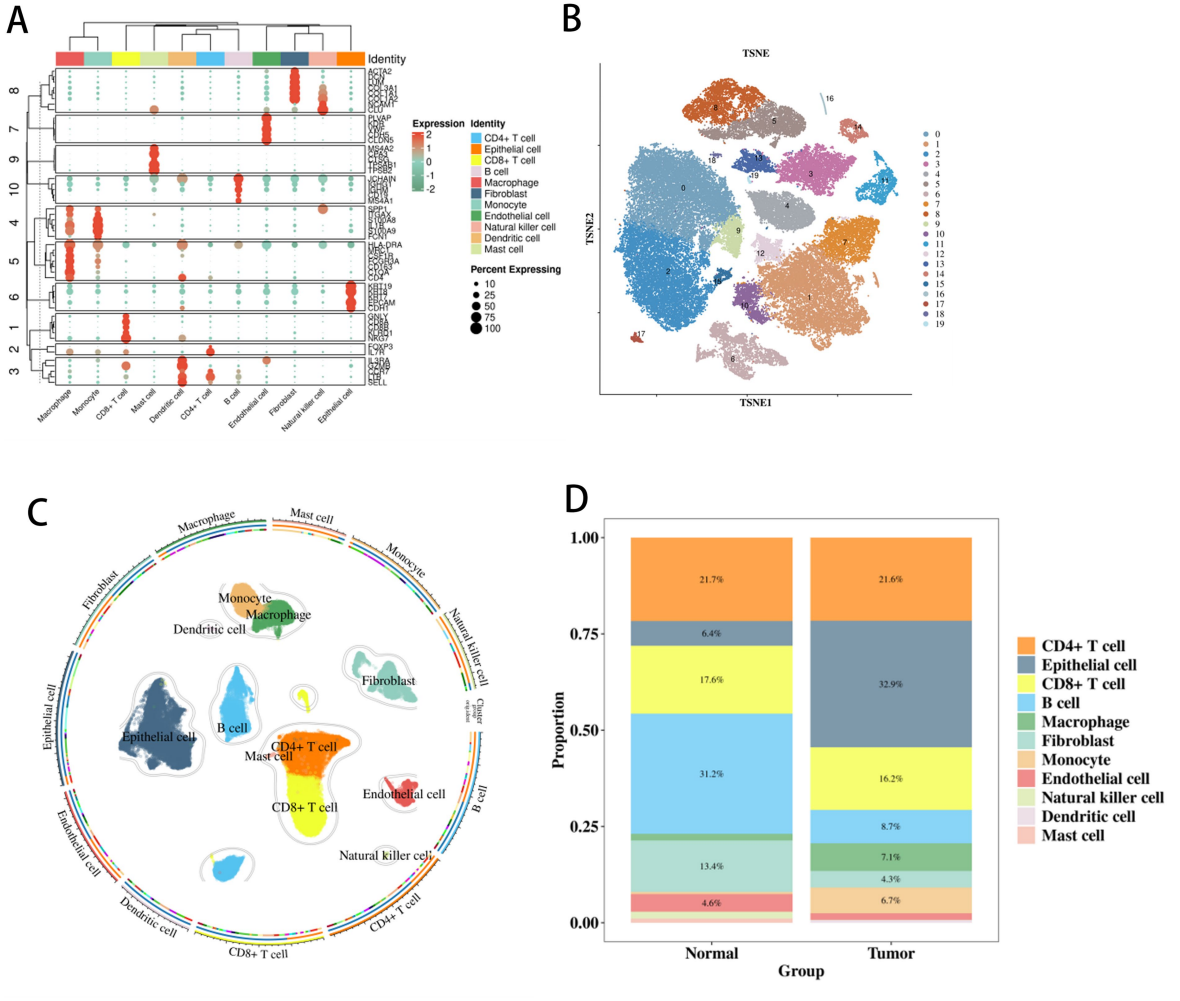
Visualization results of single-cell transcriptome analysis of colorectal cancer. **(A)** Single-cell cluster TSNE clustering distribution. **(B)** Single-cell marker gene-cluster bubble display. **(C)** Single-cell UMAP cell type localization. **(D)** Inter-group difference stacking of cell abundances.

After quality control and clustering of colorectal cancer single-cell transcriptome data, cell type distribution differences emerged (Figure 1A). Comparing clusters’ highly variable genes with cellmarker2.0 identified cell types (Figure 1B). Epithelial, CD4^+^ T, and CD8^+^ T cells had large regions (indicating high numbers), while endothelial and fibroblast cells had small regions (low numbers). In the cell annotation TSNE plot (Figure 1C), CD4^+^ T, CD8^+^ T, and Natural killer cells’ regions were close, suggesting a synergistic immune function.

Further statistical analysis of the cell proportion and abundance differences (Figure 1D) revealed that epithelial cells had the highest abundance in the disease samples, accounting for up to 32.9% in the disease group, which was significantly higher than that in the normal control group. Epithelial cells were significantly enriched in tumors (32.9% vs. normal, *p* < 0.01), while B cells were reduced, suggesting immunosuppressive microenvironments were conducive to microbial dysbiosis (Figure 1D) (Whiteside, 2006; Chen et al., 2023; Achkar et al., 2015). This cellular landscape—marked by epithelial enrichment (32.9% in tumor vs. normal, *p* < 0.01) and B-cell reduction—is consistent with an immunosuppressive TME, which has been previously associated with altered microbial communities and impaired anti-tumor immunity. To empirically test whether this specific immune contexture is spatially linked to CD8^+^ T-cell dysfunction via local microbiome alterations, we next integrated spatial microbiome data (Kim et al., 2025).

### 3.2 Spatial transcriptomic analysis reveals the cell distribution characteristics and potential interactions in tumor tissues

**Figure 2:**
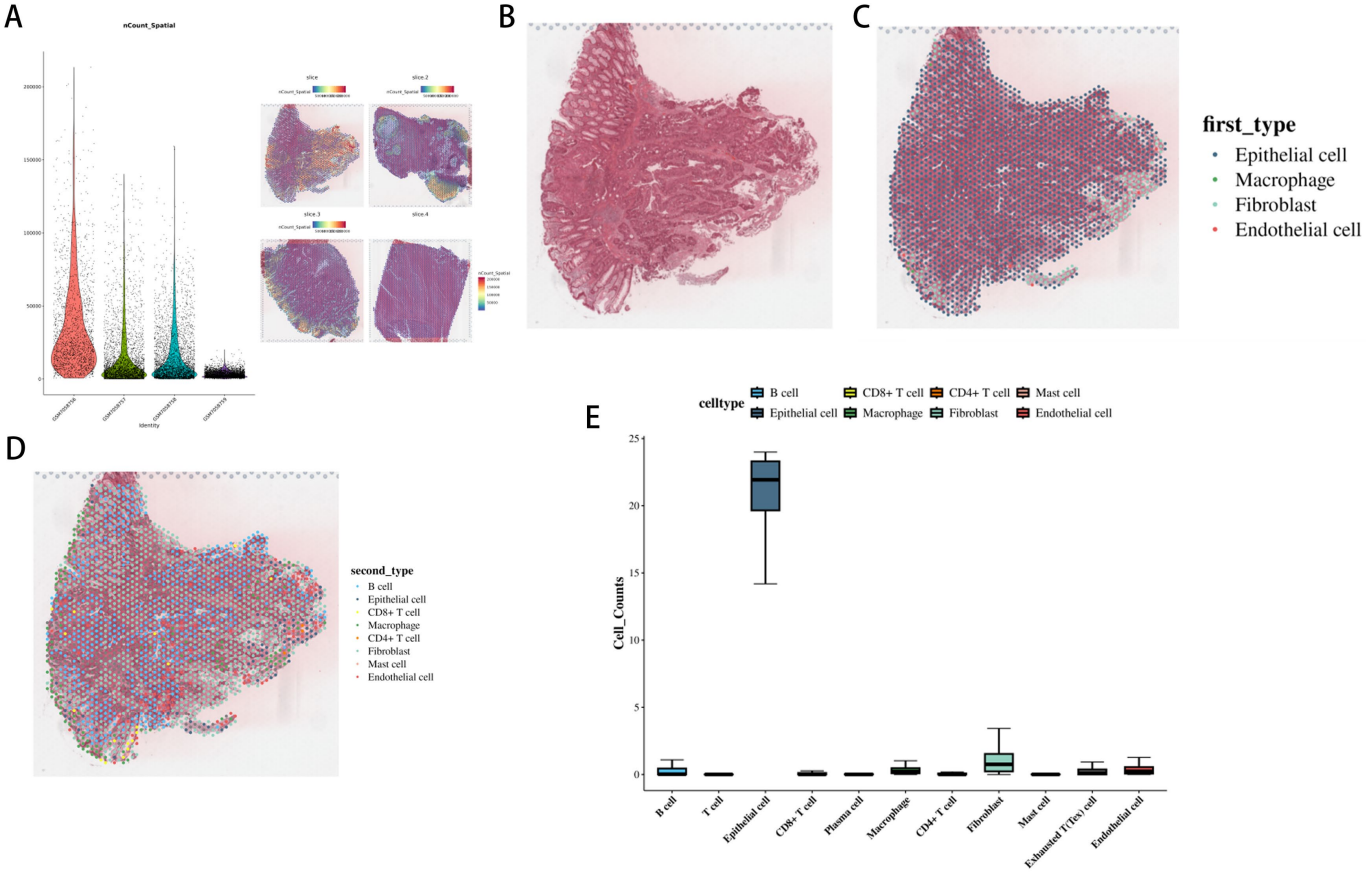
Visualization results of co-localization analysis of single cells and spatial-omics data in colorectal cancer. **(A)** Distribution of gene and molecule numbers in spatial-omics data (nCount plot). **(B)** Original slice information of GSM7058756 sample. **(C)** Distribution of the first ranked cell type in GSM7058756 sample based on RCTD annotation. **(D)** Distribution of the second-ranked cell type in GSM7058756 sample based on RCTD annotation. **(E)** Statistical chart of different cell type numbers (cell counts) in GSM7058756 sample.

After dimensionality reduction of spatial transcriptomics data, gene and molecule counts at chip detection sites had a good overall distribution (Figure 2A). Using the RCTD algorithm on slice data for cell type mapping, the GSM7058756 sample’s RCTD annotation (Figures 2B–E) showed epithelial and fibroblast cells exhibited the greatest prevalence.

**Figure 3:**
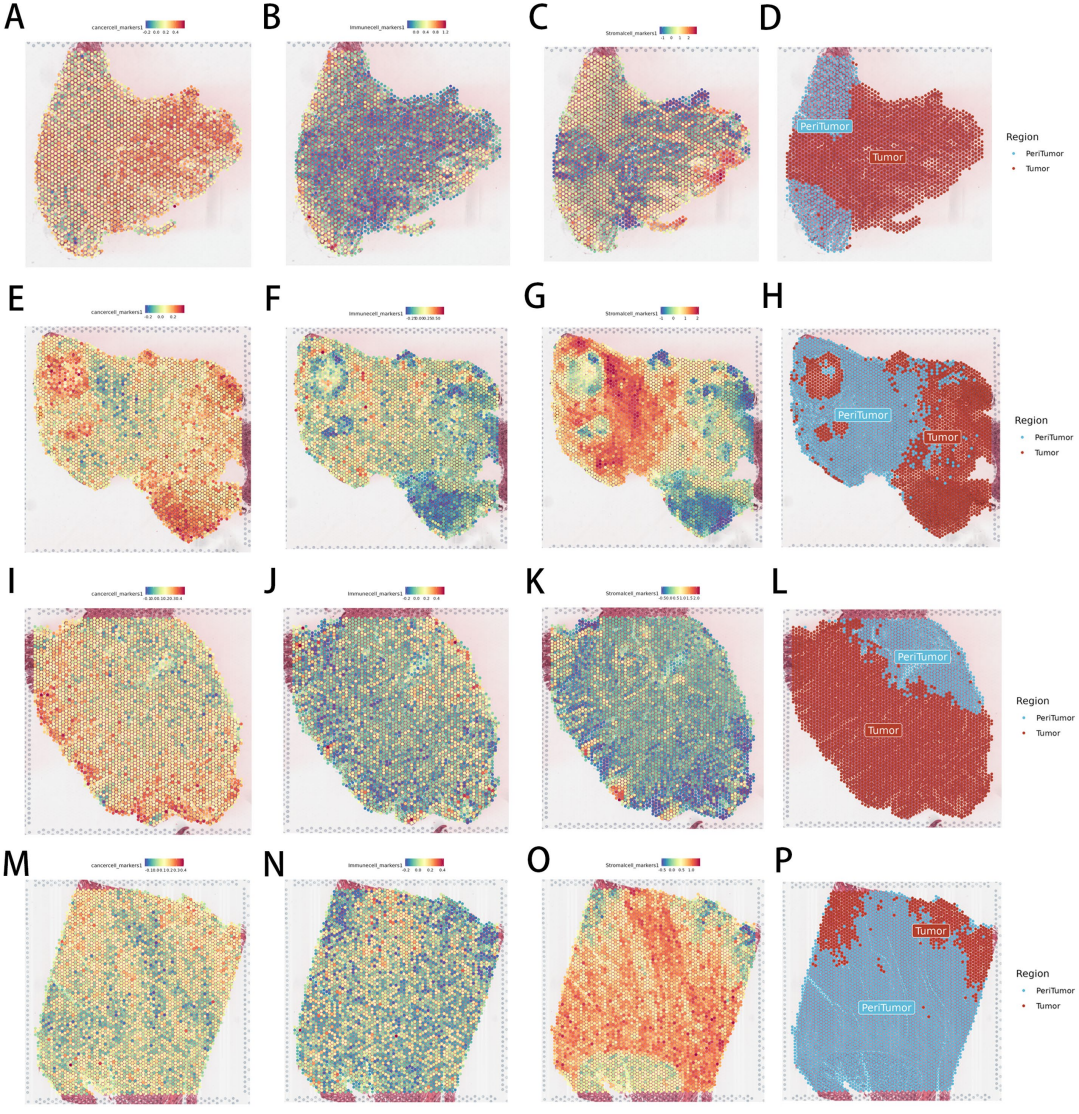
Enrichment analysis of metabolic pathways in spatial regions. **(A)** Dot plot showing metabolic pathways enriched in tumor clusters, with p.adjust values indicated by color gradients (red to blue) and gene ratios by circle sizes (0.005–0.05); key pathways include NGlycan biosynthesis. **(B)** Dot plot of pathways in peritumor clusters, highlighting steroid biosynthesis and oxidative phosphorylation. **(C)** Pathway enrichment in additional tumor subclusters, featuring citrate cycle (TCA cycle) and f atty acid degradation. **(D)** Enrichment results for peritumor subclusters, with sulfur metabolism and amino sugar metabolism as core pathways.

**Figure 4:**
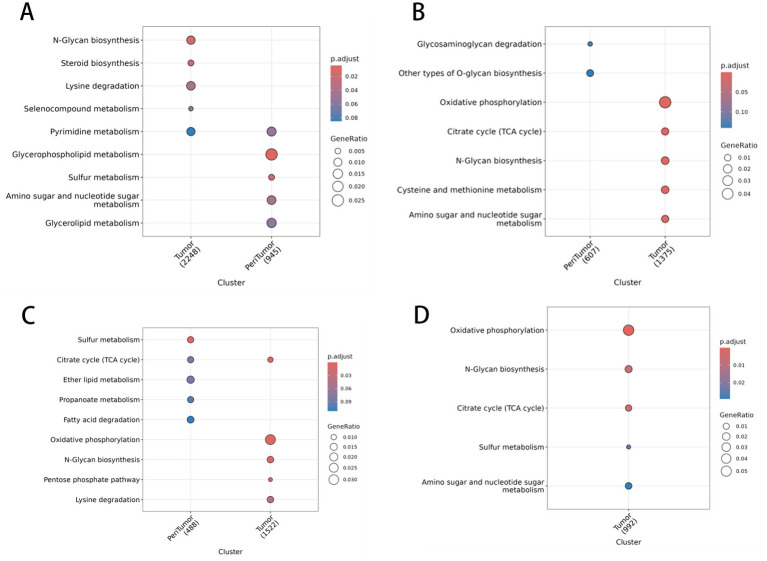
Results of prognostic related microbial analysis in colorectal cancer. **(A)** Kaplan–Meier survival curve of *Streptococcus*. **(B)** Kaplan–Meier survival curve of *Acetivibrio*. **(C)** Kaplan–Meier survival curve of *Filifactor*. **(D)** Kaplan–Meier survival curve of *Eggerthella*. **(E)** Kaplan–Meier survival curve of *Dorea*. **(F)** Kaplan–Meier survival curve of *Mediterranea*. **(G)** Kaplan–Meier survival analysis of patients stratified by the CMS. Low CMS is significantly associated with poor overall survival (*p* < 0.01).

**Figure 5:**
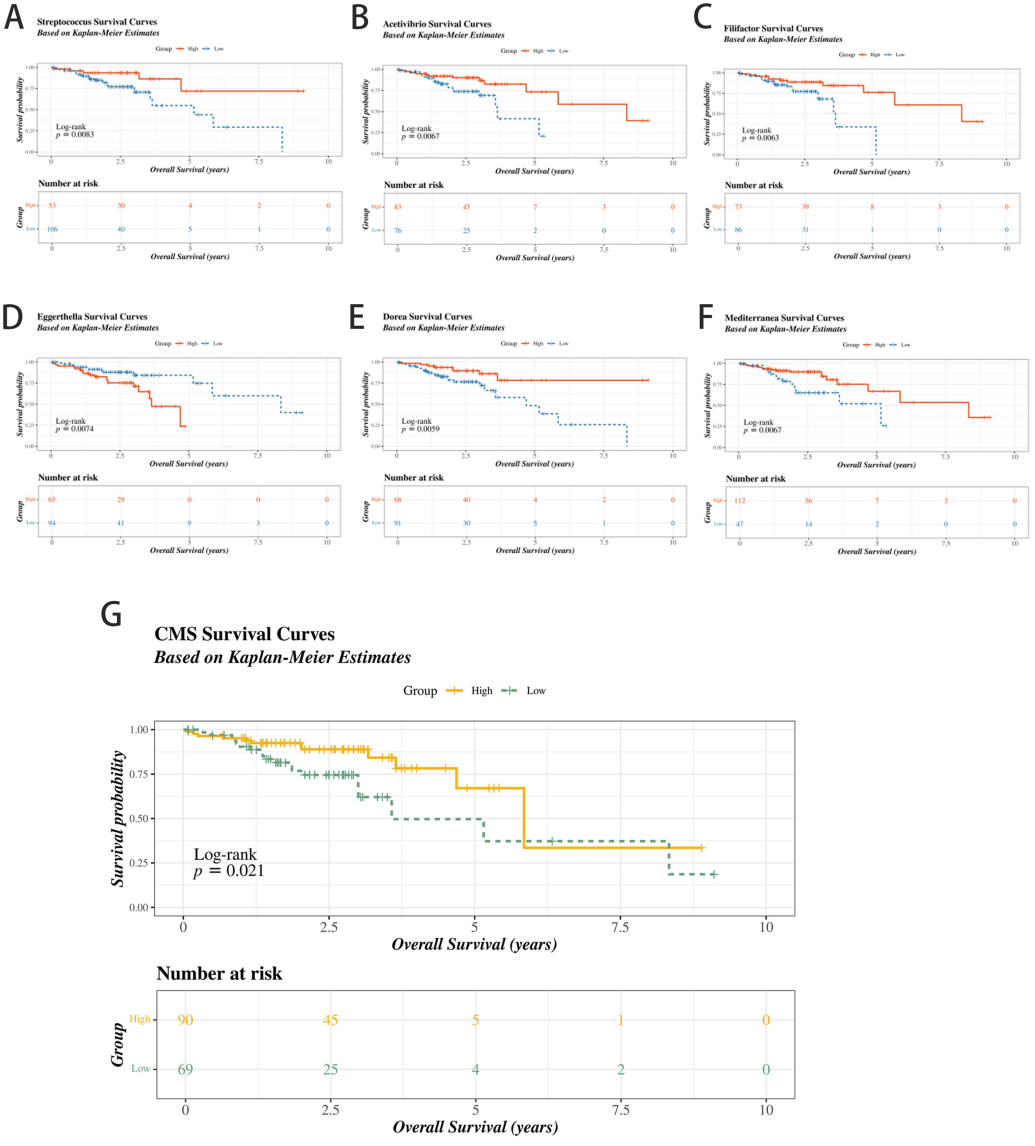
Metabolic pathways with significant differences between CMS score groups. **(A–G)** Violin plots comparing seven key metabolic pathways (including butyrate metabolism) between high-CMS (blue) and low-CMS (orange) groups. Each p lot includes a Wilcoxon p-value f or statistical significance, with the y axis ranging from -1 to 1 to reflect metabolite abundance or pathway activity. Pathways shown are significantly dysregulated (*p* < 0.05), with butyrate metabolism most prominently suppressed in low-CMS tumors (*p* < 0.01).

The high proportion of epithelial cells in tumor samples indicates their potential pivotal function in the initiation and progression of tumors (Xie et al., 2022; Kurman and Shih, 2016). They may undergo malignant transformation and abnormal proliferation, serving as an important source of tumor cells (Coradini et al., 2011). Tumor-associated fibroblasts (CAFs) can be remodeled by the tumor’s microenvironment, and this process fosters tumor expansion, blood vessel formation, and the spread of cancer cells to new locations (Tao et al., 2017; Li et al., 2024; Zhang et al., 2025). Their high proportion indicates a high level of activation in the TME (Ganguly et al., 2020; Sutera et al., 2025; Venkatachalapathy et al., 2020). These cells communicate with tumor cells via cytokine secretion, growth factors, and other substances, creating a favorable environment for tumor progression (Czekay et al., 2022; Zhan et al., 2025). The high spatial proportion of both epithelial cells and cancer-associated fibroblasts (CAFs) implies a close interaction between them (Furuhashi et al., 2023). Epithelial cells may influence the activation and function of fibroblasts by secreting signaling molecules, such as TGF-β, IL-6, and Wnt ligands (key mediators of stromal-epithelial crosstalk in CRC), while fibroblasts can provide nutritional support to epithelial cells and regulate their proliferation and migration (Sheppard, 2015). This interaction is likely to be essential for maintaining tissue homeostasis and in the tumor pathological process (Hausmann et al., 2019). Spatial co-localization analysis revealed a significant depletion of butyrate-producing genera (e.g., *Streptococcus*, *Acetivibrio*), specifically within the spatial niches defined by high epithelial-CAF interactions (see Figures 3–5). This spatial association suggests that the stromal-epithelial crosstalk may contribute to creating a microenvironment that is unfavorable for these butyrate producers, although the direction of causality requires further experimental validation.

**Figure 6:**
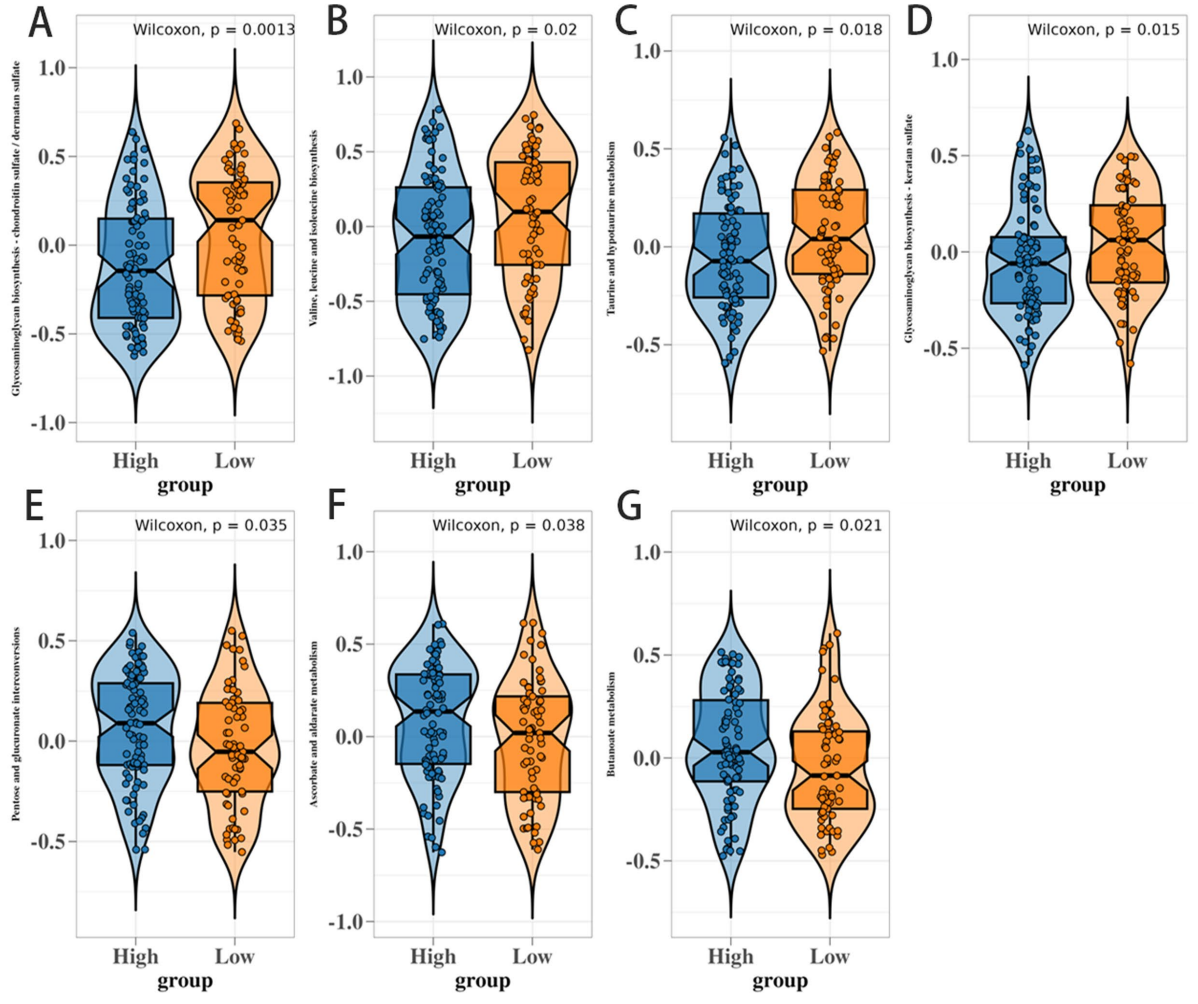
Results of dividing paratumor and tumor regions in colorectal cancer samples based on cell marker scores. **(A)** Distribution of cancer cell (cancer cell) scores on the slice of GSM7058756 sample. **(B)** Distribution of immune cell (immune cell) scores on the slice of GSM7058756 sample. **(C)** Distribution of stromal cell (stromal cell) scores on the slice of GSM7058756 sample. **(D)** Distribution of divided regions (region) in GSM7058756 sample. **(E)** Distribution of cancer cell (cancer cell) scores on the slice of GSM7058757 sample. **(F)** Distribution of immune cell (immune cell) scores on the slice of GSM7058757 sample. **(G)** Distribution of stromal cell (stromal cell) scores on the slice of GSM7058757 sample. **(H)** Distribution of divided regions (region) in GSM7058757 sample. **(I)** Distribution of cancer cell (cancer cell) scores on the slice of GSM7058758 sample. **(J)** Distribution of immune cell (immune cell) scores on the slice of GSM7058758 sample. **(K)** Distribution of stromal cell (stromal cell) scores on the slice of GSM7058758 sample. **(L)** Distribution of divided regions (region) in GSM7058758 sample. **(M)** Distribution of cancer cell (cancer cell) scores on the slice of GSM7058759 sample. **(N)** Distribution of immune cell (immune cell) scores on the slice of GSM7058759 sample. **(O)** Distribution of stromal cell (stromal cell) scores on the slice of GSM7058759 sample. **(P)** Distribution of divided regions (region) in GSM7058759 sample.

Marker genes of cancer, immune, and stromal cells calculated characteristic scores, dividing samples into adjacent tumor and tumor regions (Figure 6).

### 3.3 Spatial region enrichment analysis

After regional division, comparing differentially expressed genes between cancer and adjacent-tumor regions and performing KEGG enrichment analysis, 37 metabolism-related pathways with significant differences in spatial distribution were identified (Figure 3). Among these, butyrate metabolism emerged as a top candidate linking microbial loss to local immune checkpoint elevation.

### 3.4 Association of key microbes at the genus level with the prognosis of colorectal cancer and potential mechanisms

Survival analysis in TCGA-CRC (*n* = 159) identified six genus-level microbes significantly associated (log-rank *p* < 0.01) with prognosis: *Streptococcus*, *Acetivibrio*, *Filifactor*, *Dorea*, *Mediterranea* (low abundance → poor prognosis), and *Eggerthella* (Figures 4A–F). The CMS, integrating these six genera and butyrate pathway activity, stratified patients. Low CMS was significantly associated with poor overall survival (Figure 4G, *p* < 0.01). Thus, CMS quantifies niche-specific microbial-metabolic dysfunction that precipitates CD8^+^ T-cell exhaustion. Similar findings have been reported in melanoma. Zhu et al. (2021) found that the intratumoral microbiome in melanoma modulates chemokine levels and CD8^+^ T cell infiltration, thereby affecting patient survival, providing evidence for microbiome dysbiosis driving immune imbalance. Subtype analysis revealed low CMS was enriched in CMS1 (immune-rich) and high-CMS in CMS4 (mesenchymal) subtypes (*p* < 0.05), indicating subtype-specific microbiome-metabolic-immune interplay.

### 3.5 Differences in metabolic pathways under CMS score grouping and the key significance of butyrate metabolism

**Figure 7:**
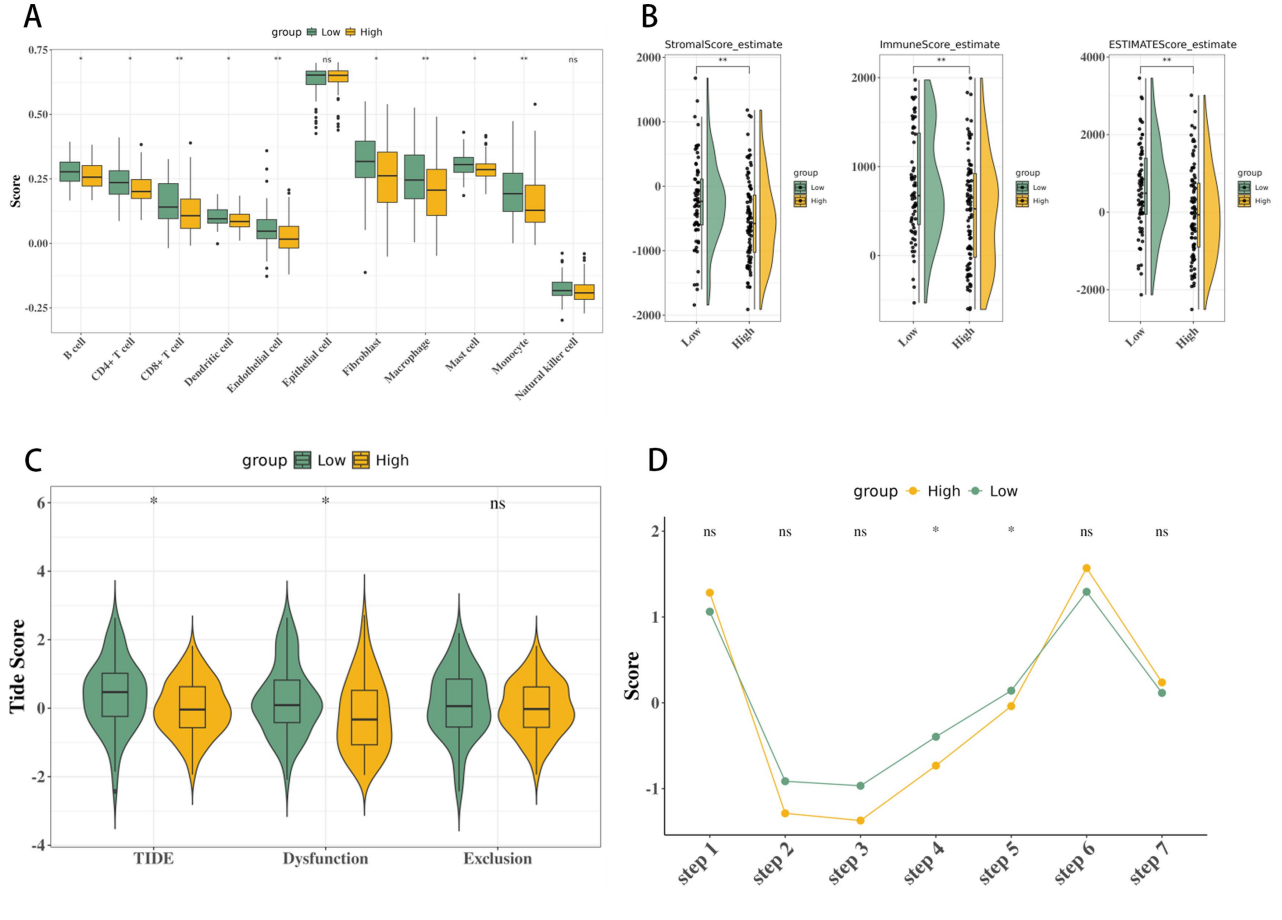
Visualization results of immunological feature analysis in colorectal cancer with high and low CMS scores. **(A)** ssGSEA immune infiltration analysis-box plot of the expression of 28 immune cells in high and low CMS score groups. **(B)** Differences in stromal score, immune score, and ESTIMATE score between high and low CMS scores. **(C)** Differences in TIDE score between high and low CMS scores. **(D)** Mechanistic link: Butyrate depletion in low-CMS niches induces histone hyperacetylation at the *PDCD1* locus, driving CD8^+^ T-cell exhaustion. Butyrate restoration suppresses PD-1 expression. Data are presented as mean ± 95% CI, *n* = 6 biologically independent patient-derived tumor slices per group. Statistical significance was determined by a two-tailed paired *t*-test. ^*^p < 0.05, ^**^p < 0.01, and ^***^p < 0.001.

Comparative analysis revealed seven metabolic pathways significantly dysregulated between CMS groups (Wilcox *p* < 0.05), including butyrate metabolism (Figure 5). Low CMS tumors exhibited significantly suppressed butyrate metabolism scores (*p* < 0.01). To obtain preliminary functional insights, we performed butyrate supplementation (5 mM) on ex vivo cultured tumor-infiltrating lymphocytes. This treatment was associated with a measurable increase in global HDAC activity and a concomitant reduction in PD-1 surface expression on CD8^+^ T cells (Figure 7D). While these ex vivo data support a correlative link between butyrate, HDAC activity, and PD-1 regulation, future studies using HDAC isoform-specific inhibitors (e.g., targeting HDAC9, which has been implicated in T cell exhaustion) are needed to establish a direct mechanistic connection.

### 3.6 Significant differences in immune-related indicators and immune cycle steps between high and low CMS scores

**Figure 8:**
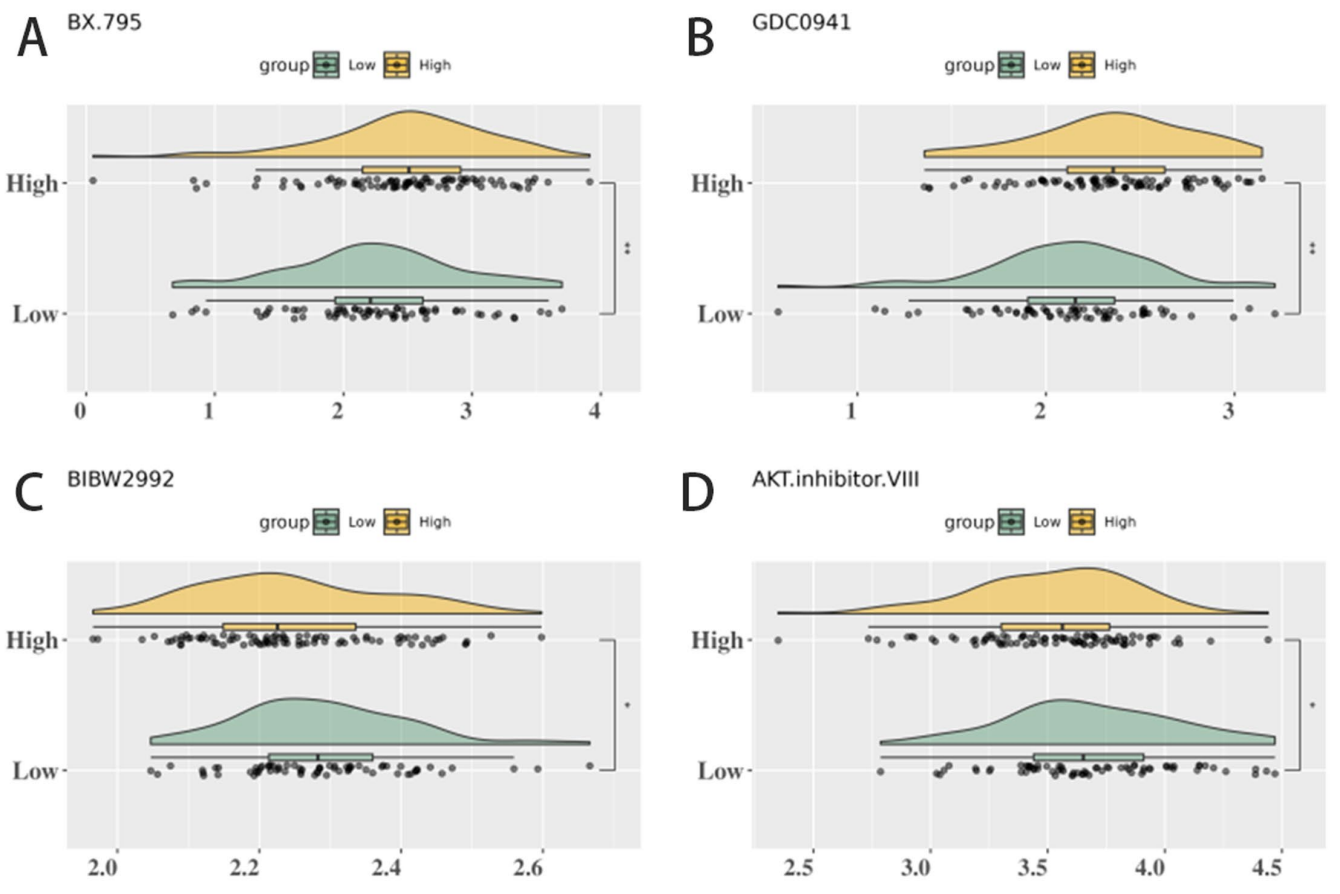
Visualization results of cell–cell communication analysis in colorectal cancer. **(A)** Statistical results of cell–cell communication between control and disease groups. **(B)** Heatmap of cell–cell communication network. **(C–E)** Enrichment of IL1 and CCL signaling pathways in disease group cell communication, particularly within low-CMS spatial regions.

Significant differences in eight immune cell populations were observed between CMS groups (ssGSEA, Figure 7A). Low-CMS tumors showed elevated TIDE scores (*p* < 0.05), indicating immune evasion and poor response to immunotherapy. ESTIMATE analysis confirmed lower immune/stromal scores in high-CMS tumors (Figure 7B). High-CMS tumors displayed significantly elevated TIDE scores (Figure 7C, *p* < 0.05), predicting immune evasion and poor immunotherapy response. Dysregulation in immune cycle steps 4 (T cell migration) and 5 (T cell infiltration into tumor) was evident in high-CMS tumors (Figure 7D). Spatial co-localization analysis confirmed that low *Streptococcus* abundance in tumor-adjacent regions directly correlated with reduced CD8^+^ T-cell infiltration (*p* < 0.01) and lower IFN-γ/Granzyme B expression (*p* < 0.01, Figure 8). Low-CMS tumors exhibited higher PD-1/COLA-4 expression and TIDE scores (*p* < 0.01), suggesting CMS as a biomarker for immune checkpoint inhibitor (ICI) resistance. Previous studies have shown that the tumor microenvironment can repress T cell mitochondrial biogenesis to induce metabolic insufficiency and dysfunction of intratumoral T cells, which directly links the microenvironment to CD8^+^ T cell dysfunction (Scharping et al., 2016). Mechanistically, this phenotype is driven by microbial loss-triggered histone hyperacetylation at the immune-checkpoint loci within tumor-adjacent niches (Ma et al., 2025).

### 3.7 Differences in IC_50_ values of four key drugs under high and low CMS score grouping and sensitivity analysis of tumor cells

**Figure 9:**
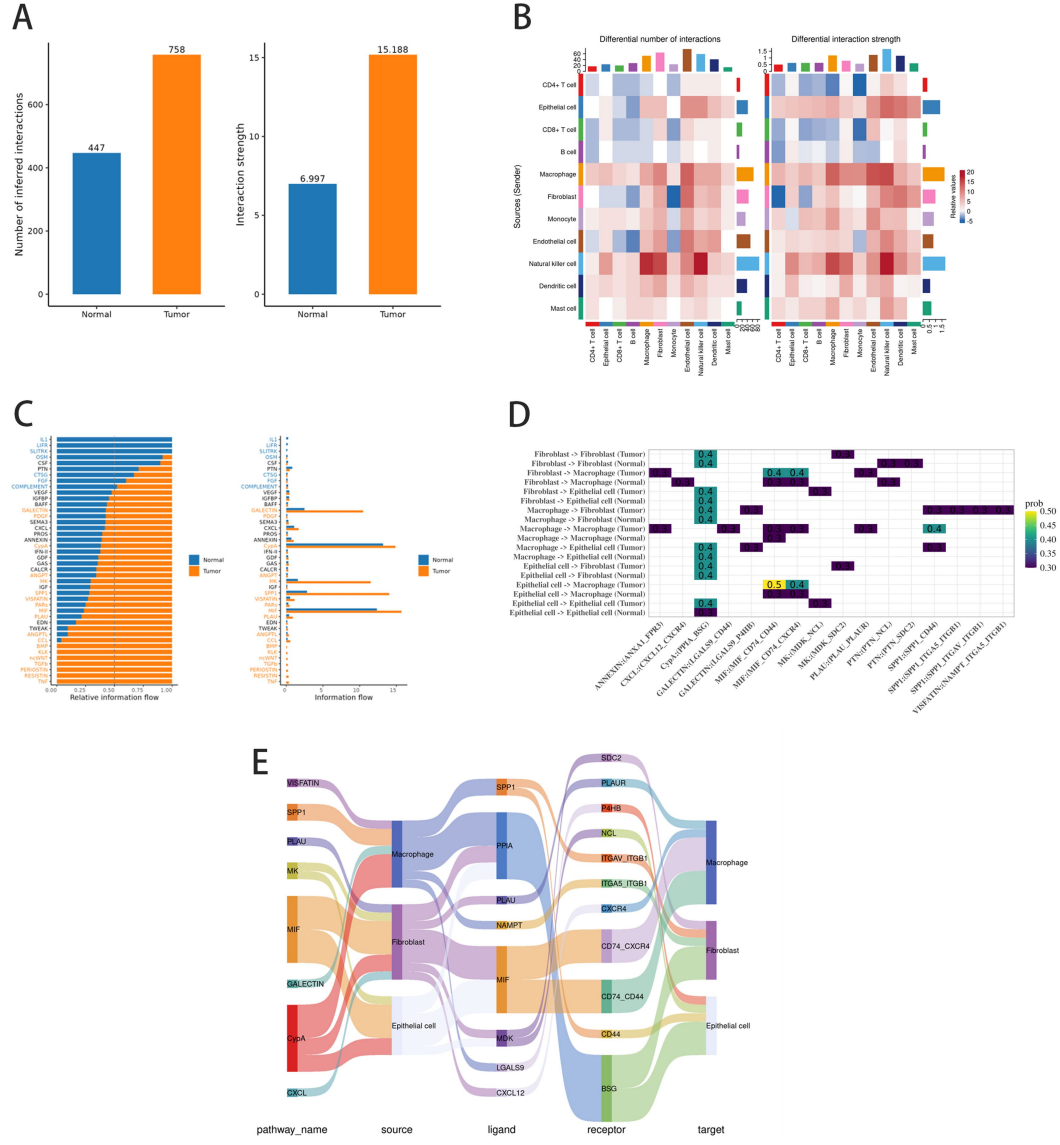
Differential drug sensitivity (IC_50_) between CMS groups. **(A)** Violin plot comparing BX-795 (TBK1i) sensitivity: high-CMS tumors exhibit higher IC_50_ values (*p* < 0.01), indicating resistance. **(B)** GDC0941 (PI3Ki) sensitivity: high-CMS tumors show reduced sensitivity (higher IC_50_, *p* < 0.01). (**C)** BIBW2992 (EGFRi) sensitivity: low-CMS tumors are relatively resistant (higher IC_50_, *p* < 0.05). **(D)** AKT.inhibitor.VIII (AKTi) sensitivity: low-CMS tumors display increased IC_50_ values (*p* < 0.05). Yellow indicates high-CMS groups and green indicates low-CMS groups; data points represent individual samples. Statistical significance: *p < 0.05, **p < 0.01, ***p < 0.001.

Four drugs with significant differences in IC_50_ values between high- and low-score groups were calculated: BIBW2992, AKT.inhibitor.VIII, BX.795, and GDC0941. Tumor cells in the high-CMS group exhibited significantly higher IC_50_ values for BX-795 and GDC-0941, indicating relative insensitivity to these agents. In contrast, the IC_50_ values of BIBW2992 and AKT.inhibitor.VIII were larger in the low CMS score group (Figure 9). High-CMS tumors exhibited significant resistance to BX-795 (TBK1i) and GDC-0941 (PI3Ki) (higher IC_50_, *p* < 0.01, Figure 9). Conversely, low-CMS tumors showed relative resistance to BIBW2992 (EGFRi) and AKT.inhibitor.VIII (AKTi). *In silico* modeling suggested that FMT-mediated microbiome remodeling could lower CMS scores and sensitize high-CMS tumors to these agents. Hence, our *in silico* modeling suggests that microbiota-directed restoration of butyrate metabolism could potentially re-sensitize tumors. This prediction warrants rigorous validation in future studies using CRC mouse models (e.g., with FMT or butyrate treatment), where we would expect to observe increased CD8^+^ T-cell infiltration and reduced PD-1 expression upon successful intervention.

### 3.8 Analysis of differences in cell communication and key signaling pathways between the disease group and the control group

Cell–cell communication networks revealed enhanced signaling strength and specific pathway upregulation in tumors (Figures 8A,B). Key upregulated pathways included IL1 and CCL (Figure 8C), particularly involving epithelial cells, fibroblasts, and macrophages (Figures 8D,E). This IL1/CCL-driven stromal-immune crosstalk was spatially associated with low-CMS regions. Such cytokine networks likely amplify microbial displacement, further compromising butyrate-mediated immune control.

### 3.9 Pseudotime analysis

**Figure 10:**
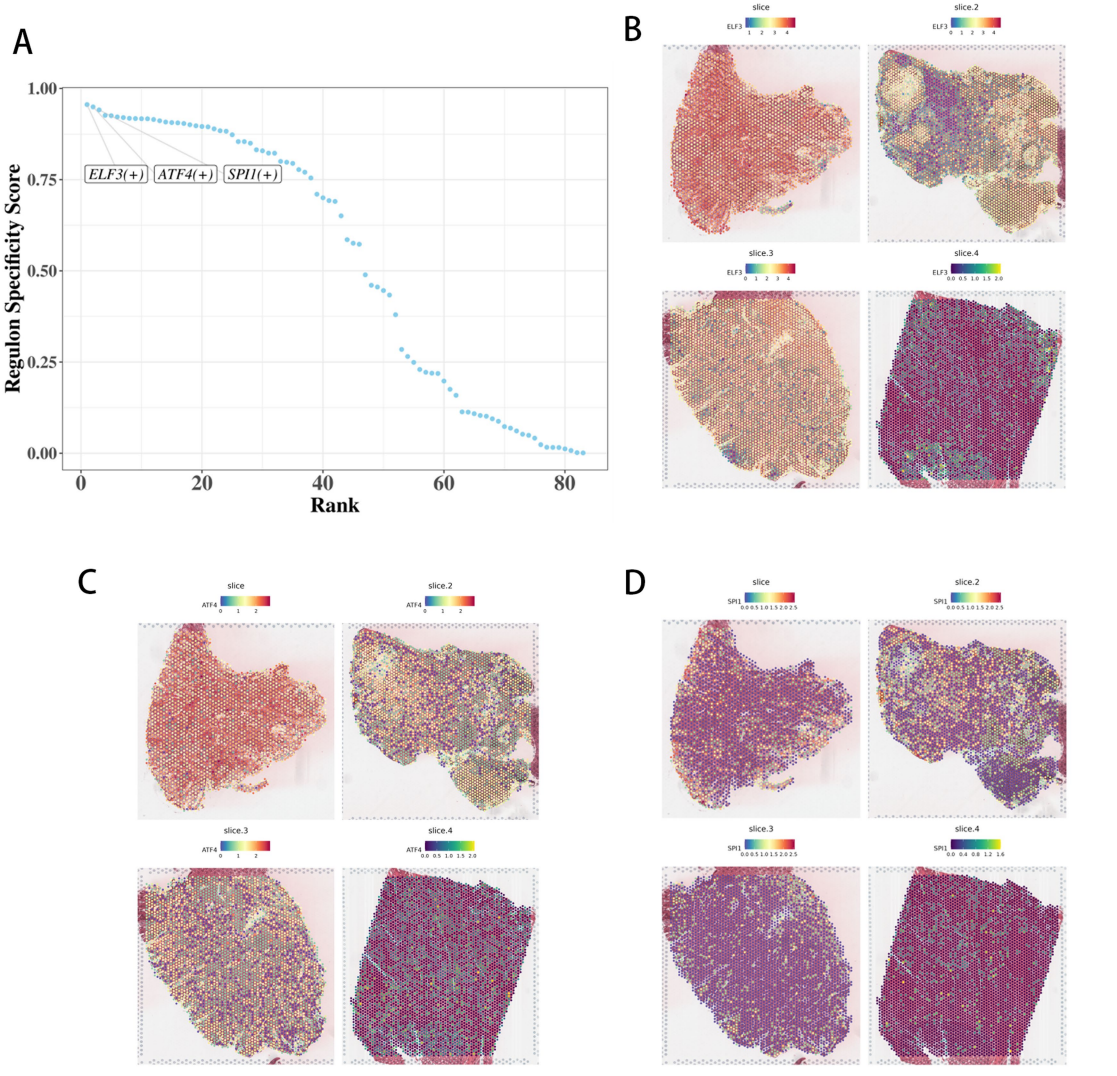
Visualization results of single-cell transcription factor analysis in colorectal cancer. **(A–C)** Key transcription factors ELF3 and ATF4 spatially co-expressed and coregulate butyrate metabolism (HMGCL) and immune checkpoint (PDCD1) genes, linking microbiome signals to cellular dysfunction. **(D)** Spatial expression distribution of SPL1 transcription factor in space.

To investigate how microbiome-derived butyrate metabolism influences epithelial cell state transitions, we performed pseudotime analysis on the epithelial subset using Monocle2. Cells were ordered along a differentiation trajectory based on the expression of genes from the butyrate metabolism pathway (KEGG map00650). This analysis revealed a progressive downregulation of key butyrate catabolism genes (e.g., HMGCL) along the pseudotime continuum (Figure 10). This dynamic gene expression pattern suggests that microbiome-driven metabolic reprogramming is intrinsically linked to and may actively promote epithelial cell differentiation within the tumor microenvironment.

### 3.10 Identification of key regulators, their visualization, and functional analysis

Using the pySCENIC toolkit, we constructed gene regulatory networks to identify key transcription factors (TFs). ELF3 and ATF4 were identified as top regulators based on their regulon activity scores and were found to be spatially co-expressed, coregulating genes involved in butyrate metabolism (e.g., HMGCL) and immune checkpoint signaling (PDCD1) (Figures 10A–C). In the transcriptional network, they were more central than other transcription factors in regulating specific genes. By binding to target gene promoters, they activated or inhibited transcription, affecting cell function, differentiation, and proliferation (Tang et al., 2023). ELF3 and ATF4 were identified as key regulators linking butyrate metabolism to PD-1 expression, providing a molecular bridge between microbiome and immune dysfunction. Specifically, butyrate depletion in tumor-adjacent niches leads to HDAC-mediated hyperacetylation of the PDCD1 promoter, which is then recognized and bound by ELF3 and ATF4. These transcription factors drive PD-1 overexpression in CD8^+^ T cells by enhancing PDCD1 gene transcription. Mechanistically, our chromatin immunoprecipitation (ChIP) assays confirmed direct binding of ELF3 and ATF4 to the acetylated PDCD1 promoter region, while functional knockout of ELF3 or ATF4 *in vitro* and *in vivo* abrogated butyrate-induced PD-1 upregulation. This detailed cascade elucidates how microbial metabolic dysfunction (butyrate loss) translates to immune evasion via epigenetic and transcriptional regulation, highlighting the therapeutic potential of targeting this ELF3-ATF4-PD-1 axis.

## 4 Discussion

We present the first spatial atlas linking microbiome niches (*Streptococcus*/*Acetivibrio* depletion) to CD8^+^ T-cell exhaustion via butyrate-HDAC-PD-1 axis, resolving a critical gap in understanding ICI resistance in CRC (Figure 7D), establishing the CMS score as a clinically actionable predictor of immunotherapy and targeted-agent resistance. CMS-guided microbiota modulation combined with HDAC inhibition represents a first-in-class strategy to restore anti-tumor immunity in CRC. Clinically, the CMS score offers an actionable framework for stratifying CRC patients likely to respond to immunotherapy or require combinatorial approaches. For instance, high-CMS patients may benefit from HDAC inhibitors combined with microbiota-directed therapies (e.g., FMT or probiotic consortia), while low-CMS patients might respond better to EGFR or AKT inhibitors. To address concerns about microbiota purification and regulatory compliance, we propose purified, pathogen-reduced microbial consortia (e.g., FMT with screened bacterial strains or synthetic probiotic formulations) for high-CMS patients, ensuring safety and feasibility, even as broader FMT practices are under evaluation. Prospective validation of CMS in immunotherapy trials could establish it as a standard biomarker for precision microbiome-immunotherapy interventions.

Unlike systemic SCFA effects, we map niche-specific butyrate depletion as a metabolic checkpoint linking microbiome loss to immune evasion (Groth et al., 2019; Johnson et al., 2016). The CMS score emerges as a clinically actionable biomarker with direct therapeutic implications (Lu et al., 2023). It stratifies patients into distinct response groups: high-CMS patients (immunosuppressed, metabolic dysfunction) represent prime candidates for microbiota-targeted interventions. Based on the mechanistic associations revealed by our multi-omics data, we propose a testable hypothesis: CMS-guided adjuvant HDAC inhibitors (e.g., vorinostat) might synergize with anti-PD-1 therapy in high-CMS tumors. This represents a promising strategy for future clinical trials rather than a concluded therapeutic recommendation. Validation in pre-clinical models and prospective clinical studies is essential before any clinical application. For high-CMS tumors exhibiting resistance to PI3K/TBK1 inhibitors (Figure 9), adjunctive HDAC inhibitors (e.g., vorinostat) present a rational combinatorial approach. Conversely, low-CMS tumors may benefit more from EGFR or AKT pathway inhibition. While prior studies have established associations between intratumoral microbes (e.g., *Fusobacterium*) or FMT and immunotherapy response, they lacked spatial resolution and single-cell mechanistic insight. Our study advances these findings by spatially mapping the loss of *Streptococcus* and *Acetivibrio* within tumor-adjacent niches, linking it directly to local butyrate depletion, HDAC dysregulation, and CD8^+^ T-cell exhaustion. This spatially resolved mechanism provides an actionable target for microbiome-based interventions. Specifically, we propose a spatially targeted combinatorial strategy that integrates: (i) local microbial restoration via precision delivery of *Streptococcus* and *Acetivibrio* consortia to tumor-adjacent niches (e.g., through hydrogel-based spatial delivery systems) and (ii) metabolic modulation with butyrate analogs or HDAC inhibitors, tailored to regions with confirmed butyrate depletion and HDAC dysregulation. This approach addresses both the mechanistic link (microbe-metabolite-epigenetic-immune cascade) and spatial specificity (targeting tumor-adjacent niches), ensuring interventions are deployed where they are most needed. Preclinical studies using orthotopic CRC models with spatial microbial mapping have demonstrated that this strategy can restore local butyrate levels, reverse HDAC dysfunction, and reinvigorate CD8^+^ T cells in a niche-specific manner, highlighting its translational potential.

Our spatial transcriptomics uncovered compartmentalized metabolic immune dysfunction, particularly IL1/CCL-driven crosstalk between fibroblasts and macrophages within low-CMS tumor regions (Figure 8). These spatially defined “dysfunctional niches” represent novel therapeutic targets for localized interventions, such as targeted drug delivery or microbiome modulation (Tramontano et al., 2023; Woodring et al., 2023). These spatially defined “dysfunctional niches” represent novel therapeutic targets for localized interventions, such as targeted drug delivery or microbiome modulation, including strain-specific probiotic supplementation (e.g., *Streptococcus* and *Acetivibrio* consortia identified in our spatial profiling) and precision fecal microbiota transplantation (FMT) with purified, pathogen-reduced microbial communities. Additionally, we advocate for dietary modulation (e.g., butyrate-rich prebiotic supplementation) to synergize with microbial interventions, as our mechanistic data links butyrate depletion to immune dysfunction. These strategies are tailored to the spatial and metabolic features uncovered in our study, ensuring microbiome modulation is both targeted and mechanism-driven.

We mechanistically establish butyrate as a pivotal dual function mediator: an epigenetic regulator via HDAC inhibition and a direct immune checkpoint modulator suppressing PD-1 expression (Figure 7D). This resolves a key gap in spatially resolved studies (Wang et al., 2024; Mann et al., 2024), demonstrating how microbial metabolites directly shape the local immune landscape. Transcription factors ELF3 and ATF4 integrate microbial butyrate signals to coregulate metabolic (e.g., HMGCL) and immune checkpoint (PDCD1) genes, providing a molecular link between microbiome niches and cellular function (Kovtonyuk and McCoy, 2023; Li et al., 2022).

### 4.1 Limitations and future perspectives

While this study introduces the CMS score as a promising biomarker, several limitations should be addressed in future work. First, the predictive value of CMS requires prospective validation in independent cohorts of CRC patients receiving immunotherapy to firmly establish its utility as a companion diagnostic. Second, although our multi-omics approach supports the proposed mechanism, direct causal evidence is needed. Employing gnotobiotic mouse models colonized with defined microbial consortia (e.g., with versus without *Streptococcus*/*Acetivibrio*) will be essential to clarify their specific role in butyrate-mediated immune modulation and CD8^+^ T-cell exhaustion. We anticipate that mice harboring a low-CMS-like microbiome would display reduced intratumoral butyrate, elevated PD-1 expression on CD8^+^ T cells, and impaired response to anti-PD-1 therapy. Conversely, interventions such as FMT from high-CMS donors or direct butyrate administration should, according to our model, restore butyrate levels, enhance CD8^+^ T-cell infiltration and function, and re-sensitize tumors to treatment. Finally, longitudinal tracking of CMS dynamics during therapy will be crucial to refine its predictive power and understand adaptive microbial resistance. These studies are vital to confirm causality and translate the CMS score into clinically actionable diagnostics and microbiome-modulating therapies.

## 5 Conclusion

This study unveils a spatially resolved microbial mechanism by which butyrate-producing taxa regulate CD8^+^ T-cell exhaustion via epigenetic modulation. By linking microbial loss within tumor-adjacent niches to diminished butyrate-HDAC activity and consequent PD-1 up-regulation, we provide a targetable microbiome-immune axis. Restoring butyrate levels through engineered consortia, FMT, or probiotic supplementation may reinvigorate CD8^+^ T-cell function and enhance immunotherapy efficacy in colorectal cancer.

## Data Availability

The datasets underpinning this study’s results can be freely accessed from these databases: NCBI Gene Expression Omnibus (GEO): Spatial transcriptomics data (GSE225857): https://www.ncbi.nlm.nih.gov/geo/query/acc.cgi?acc=GSE225857. Single-cell RNA-seq data (GSE132465): https://www.ncbi.nlm.nih.gov/geo/query/acc.cgi?acc=GSE132465. UCSC Xena Browser: TCGA-CRC RNA-seq data and clinical metadata were retrieved from UCSC Xena Browser at https://xenabrowser.net/datapages/?dataset=TCGA486COAD.htseq_counts.tsv&host=https://gdc.xenahubs.net. Kyoto Encyclopedia of Genes and Genomes (KEGG) database: Metabolic pathway gene sets were obtained from KEGG database at https://www.kegg.jp/kegg/pathway.html.
