# Supplementary material for: Spatial microbiome-metabolic crosstalk drives CD8+ T-cell exhaustion through the butyrate-HDAC axis in colorectal cancer
Source: Front Microbiol. 2025 Dec 8;16:1704491. doi: 10.3389/fmicb.2025.1704491 (PMC12719255; doi:10.3389/fmicb.2025.1704491)
Supplement: Supplementary file 1 [file Data_Sheet_1.pdf]

Supplementary Table S1: Marker genes used for cell type annotation in single-cell RNA-seq analysis

Cell Type	Marker Genes	Expression Criteria
Epithelial cells	EPCAM, KRT18, KRT19	High expression in epithelial cell clusters
CD4+ T cells	CD3D, CD3E, CD4, IL7R	Specific expression in T helper populations
CD8+ T cells	CD3D, CD3E, CD8A, CD8B, GZMB	Cytotoxic T cell markers
Fibroblasts	COL1A1, COL1A2, DCN, LUM, ACTA2	Stromal cell markers
Endothelial cells	PECAM1, VWF, CDH5, CLDN5	Vascular endothelial markers
Monocytes	CD14, CD68, FCGR3A	Myeloid lineage markers
Macrophages	CD68, CD163, MRC1, CSF1R	Tissue macrophage markers
Dendritic cells	CD1C, FCER1A, CLEC9A, XCR1	Antigen presenting cells
Mast cells	TPSAB1, CPA3, KIT, HDC	Mast cell-specific proteases
B cells	CD19, CD79A, MS4A1, CD22	B lymphocyte markers
Natural killer cells	NKG7, GNLY, NCAM1, KLRD1	Cytotoxic innate lymphocytes
